# Epidermal growth factor receptor mutations and brain metastases in non-small cell lung cancer

**DOI:** 10.3389/fonc.2022.912505

**Published:** 2022-11-15

**Authors:** Wei Zhao, Wei Zhou, Li Rong, Mao Sun, Xing Lin, Lulu Wang, Shiqiang Wang, Ying Wang, Zhouguang Hui

**Affiliations:** ^1^ Department of Radiation Oncology, Chongqing University Cancer Hospital and Chongqing Cancer Institute and Chongqing Cancer Hospital, Chongqing, China; ^2^ Department of Gastroenterology, Bishan Hospital of Chongqing medical university/Bishan Hospital of Chongqing, Chongqing, China; ^3^ Department of Neurosurgery, Chongqing University Cancer Hospital and Chongqing Cancer Institute and Chongqing Cancer Hospital, Chongqing, China; ^4^ Department of Radiation Oncology, National Cancer Center/National Clinical Research Center for Cancer/Cancer Hospital, Chinese Academy of Medical Sciences and Peking Union Medical College, Beijing, China; ^5^ Department of VIP Medical Services, National Cancer Center/National Clinical Research Center for Cancer/Cancer Hospital, Chinese Academy of Medical Sciences and Peking Union Medical College, Beijing, China

**Keywords:** non-small cell lung cancer, EGFR mutation, brain metastasis, genetic heterogeneity, characteristics, prognosis

## Abstract

Studies have revealed that non-small cell lung cancer (NSCLC) with epidermal growth factor receptor (EGFR) mutations has a high incidence of brain metastases (BMs). However, the association between EGFR mutations and BMs remains unknown. This review summarizes detailed information about the incidence of BMs, clinical and imaging characteristics of BMs, brain surveillance strategies, influence of treatments on BMs, prognosis after BMs, and differences in EGFR mutations between paired primary tumors and BMs in EGFR-mutated NSCLC. The prognostic results demonstrate that patients with mutated EGFR have a higher incidence of BMs, EGFR tyrosine kinase inhibitors (EGFR-TKIs) (afatinib and osimertinib) delay the development of BMs, and patients with mutated EGFR with synchronous or early BMs have better overall survival after BMs than those with wild-type EGFR. The EGFR mutation status of BM sites is not always in accordance with the primary tumors, which indicates that there is heterogeneity in EGFR gene status between paired primary tumors and BMs. However, the EGFR gene status of the primary site can largely represent that of BM sites. Among patients developing synchronous BMs, patients with mutated EGFR are less likely to have central nervous system (CNS) symptoms than patients with wild-type EGFR. However, the possibility of neuro-symptoms is high in patients with metachronous BMs. Patients with mutated EGFR tend to have multiple BMs as compared to patients with wild-type EGFR. Regarding very early-stage NSCLC patients without neuro-symptoms, regular neuroimaging follow-up is not recommended. Among advanced NSCLC patients with EGFR mutation, liberal brain imaging follow-up in the first several years showed more advantages in terms of cost.

## Introduction

In recent years, accompanied by dramatic improvements in systemic disease control and advancements in brain imaging, the incidence of non-small cell lung cancer (NSCLC) brain metastases (BMs) has increased. Patients with mutated EGFR have a much higher possibility of BMs than those with wild-type EGFR. Previous studies have reported that the 3-year cumulative rate of BMs ranges from 29.4% to 60.3% in patients with mutated EGFR and from 22% to 28.2% in patients with wild-type EGFR (1–7). Brain metastases from NSCLC are associated with substantial mortality. The median overall survival (OS) decreases significantly after the development of BMs (7), and the management of BMs is a difficult clinical problem. This is a descriptive review of the literature. The purpose was to discuss the incidence of BMs, molecular characteristics of EGFR mutation, clinical and imaging characteristics of BMs, brain surveillance strategies, influence of treatments on BMs, and prognosis after BMs among NSCLC patients, which allows us to better understand the association between EGFR mutations and BMs, inform clinical practice, and help manage brain metastases.

The followings are definitions of some terms: overall survival was defined as the survival time from the diagnosis of NSCLC until death. Overall survival after brain metastasis (BMOS) was defined as the survival time from the diagnosis of BMs to death. Synchronous BMs indicated those BMs developed at the diagnosis of NSCLC. Metachronous BMs were the BMs developed during the treatment, excluding synchronous BMs. Overall BMs were characterized by BMs that developed from diagnosis of NSCLC until death, which included synchronous and metachronous BMs. Cumulative rates (CRs) of metachronous BMs were the cumulative rates/incidences of new BMs during treatment for patients who did not have initial BMs at diagnosis. Rates/incidences of overall BMs were the proportion of BMs that occurred from the NSCLC diagnosis to death, which included both synchronous and metachronous BMs. Median time to brain metastasis (TTBM) was defined as the median time interval between the diagnosis of lung cancer and the detection of metachronous BMs.

## Incidence of brain metastases

**Figure 1:**
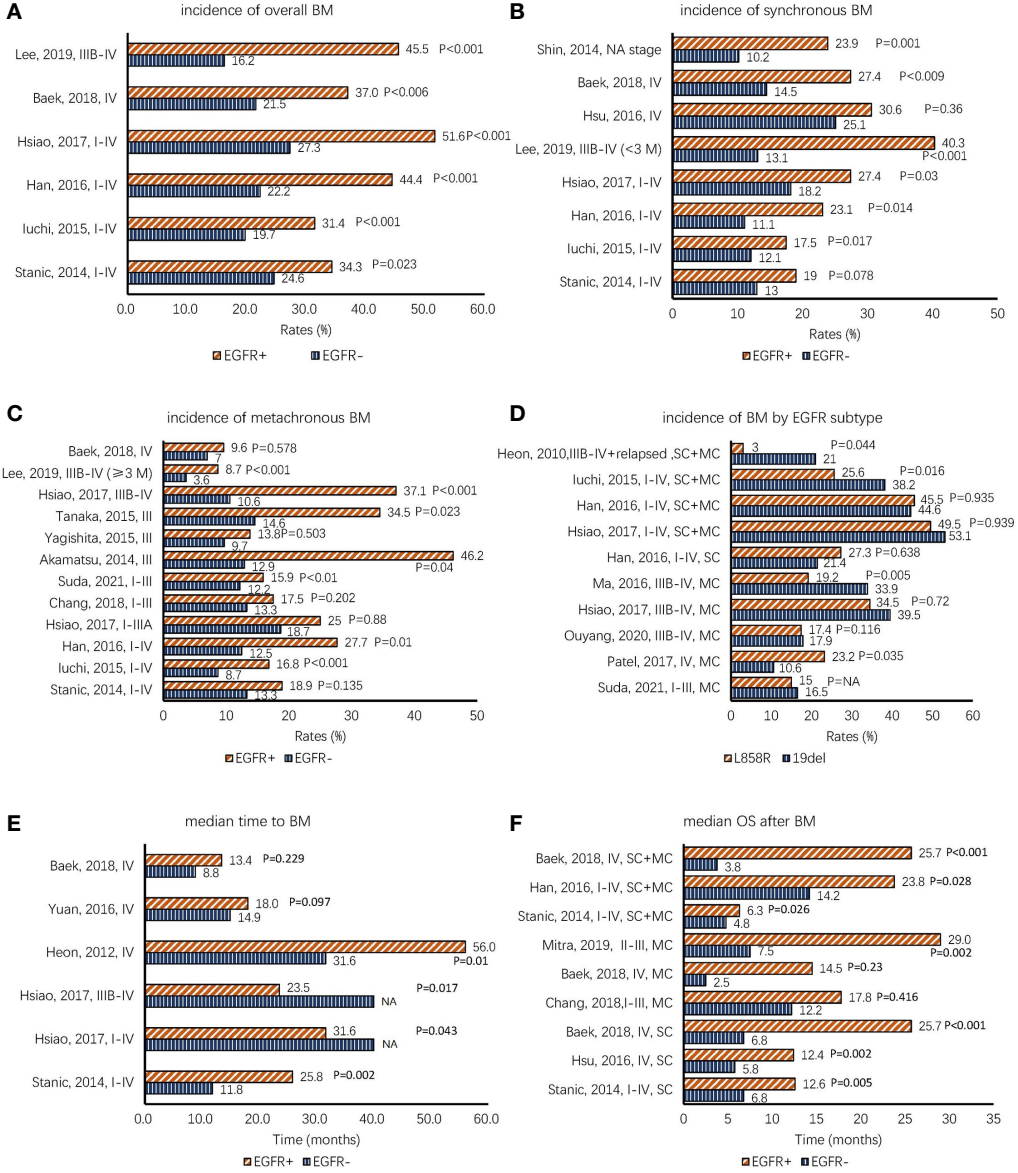
**(A–C)** Comparison of the incidence of NSCLC BMs between EGFR+ (means EGFR mutation-positive) and EGFR− (means EGFR mutation-negative) patients according to overall BMs, synchronous BMs, and metachronous BMs. **(D)** Comparison of the incidence of BMs based on EGFR mutation subtype. **(E)** The data of median time to BM, which demonstrates that EGFR-TKIs have preventive effects against BMs. **(F)** The overall survival data after BMs (BMOS); most studies demonstrate that patients with mutated EGFR have better BMOS. The items in the vertical axis mean “author, published year, stage” in panels **(A–E)** and mean “author, published year, stage, metastatic type” in panels **(D, F)**. Note. BMs, brain metastases; SC, synchronous BMs; MC, metachronous BMs; SC+MC, overall BMs; NSCLC, non-small cell lung cancer; EGFR, epidermal growth factor receptor; TKIs, tyrosine kinase inhibitors; NA, not available.

EGFR-mutated NSCLC is more prone to develop BMs than EGFR-wild NSCLC. The 3-year cumulative rate of BMs ranges from 29.4% to 60.3% in patients with mutated EGFR and from 22% to 28.2% in patients with wild-type EGFR (1–7). Rangachari et al. (5) reported that in patients still living with advanced EGFR-mutated NSCLC, BM incidence increased over time: 34.2% at 1 year, 38.4% at 2 years, 46.7% at 3 years, 48.7% at 4 years, and 52.9% at 5 years. Previous studies drew a similar conclusion that patients with mutated EGFR were more likely to have higher overall BMs than patients with wild-type EGFR regardless of the stage (**Figure 1A**) (2, 8–12). Hsu et al. (3) estimated the CRs of the overall BMs between patients with mutated EGFR and patients with wild-type EGFR among stage IV NSCLC. The cumulative rate curves were clearly separated (1-year CRs, 39% vs. 28.1%, p = 0.041; 3-year CRs, 39.2% vs. 28.2%, p = 0.038). A further subgroup analysis demonstrated that the difference in 3-year CRs was statistically significant in patients aged <66 years (59.4% vs. 35.4%), but not in patients aged ≥66 years (19.7% vs. 21.9%). Han et al. (2) reported that among stage I–IV patients, 1-, 2-, and 3-year cumulative rates of BMs were 15%, 37.7%, and 53.3% in patients with mutated EGFR, respectively, and 4.2%, 18.7%, and 22% in patients with wild-type EGFR, respectively (p = 0.001). A study by Lee et al. (8) analyzed the CRs of subsequent brain metastases (≥3 months from disease diagnosis) among 22,458 advanced-stage (stage IIIB–IV) NSCLC patients without initial brain metastasis. The CRs of BMs were significantly higher in the EGFR-positive group than in the EGFR-negative group (1-year CRs, 8.7% vs. 3.8%; 3-year CRs, 17.2% vs. 5.0%; p < 0.001).

We further discussed BM incidence among different types of BMs. **Figure 1B** demonstrates that the difference in BM incidence is significant in synchronous BMs between patients with mutated EGFR and those with wild-type EGFR (2, 3, 8–13). The risk of brain metastases increased significantly in patients with mutated EGFR at the time of diagnosis, whereas no relationship was observed between EGFR mutation status and extracranial metastases (adjusted odds ratio = 1.73, p = 0.079) (13). Experiments indicated that EGFR plays important roles in cell migration and invasion through phosphoinositide 3-kinase/protein kinase B and phospholipase C γ downstream pathways (10, 14, 15).

However, there is a discrepancy in the association between EGFR mutations and metachronous BMs (**Figure 1C**) (2, 8–12, 16–20). When considering the studies that did not show statistical significance concerning metachronous BMs, most patients were at a relatively early stage (10, 17, 20), except for two studies with stage IV patients (9, 12). In a large retrospective study in Korea (21), the diagnostic yield of the staging brain magnetic resonance imaging (MRI) was very low (0.3% in clinical stage IA disease, 3.8% in IB, and 4.7% in II). The diagnostic yield was higher in patients with adenocarcinoma (13.6%; 176 of 1,297) than in squamous cell carcinoma (5.9%; 21 of 354) (p = 0.001) and EGFR-mutant adenocarcinoma (17.5%; 85/487) than in EGFR wild-type adenocarcinoma (10.6%; 68/639) (p = 0.001). There was no statistical significance in BM rates of stage I and II patients between EGFR mutation-positive adenocarcinoma and EGFR mutation-negative adenocarcinoma (stage I, 1.8% (5/275) *vs.* 0.9% (3/335); stage II, 3.6% (1/28) *vs.* 6.9% (3/58)). In another retrospective study that enrolled patients with stage I–III NSCLC who underwent surgery, Suda et al. revealed that patients with mutated EGFR experienced postoperative brain metastases more frequently than patients with wild-type EGFR, and this difference was statistically significant in all stages except for pStage IA (pStage IA, p = 0.08; pStage IB, p < 0.001; pStage II, p = 0.02; and pStage III, p = 0.01) (19). This controversy may have arisen from differences in patient number and selection criteria, follow-up time, or intervention treatments. Hsiao et al. (10) showed that EGFR tyrosine kinase inhibitor (EGFR-TKI) was related to metachronous BMs. After the failure of the first-line treatment, the incidence of brain metastasis increased in patients with mutated EGFR compared to that of patients with wild-type EGFR (8), and acquired resistance mutations likely explain this phenomenon (22).

The influence of subtypes of EGFR mutations on the development of BMs is controversial as well. In different studies, the BM risks of patients with 19del and L858R mutation were inconsistent (**Figure 1D**) (1, 2, 7, 10, 11, 19, 23, 24). Ma et al. (24) investigated risk factors of the new central nervous system (CNS) progression in patients with stage IIIB/IV lung adenocarcinoma based on the subtype of EGFR mutations. Multivariate analysis (MA) demonstrated that L858R mutation was an independent risk factor for metachronous BMs (hazard ratio (HR) = 2.769, 95% confidence interval (CI): 1.355–5.659; p = 0.005). The work by Zhou et al. (25) and Patel et al. (23) obtained similar results that advanced NSCLC patients with L858R mutation were found to have a higher risk of developing metachronous CNS metastasis as compared to patients with 19del (Zhou: univariate analysis (UA), HR = 3.417, 95% CI: 1.653–7.064), p = 0.001; MA, HR = 3.337, 95% CI: 1.614–6.900, p = 0.001) (Patel: UA, HR = 1.93, 95% CI: 1.05–3.36, p = 0.035; MA, HR = 1.82, 95% CI: 0.99–3.36, p = 0.055). Nevertheless, Heon (1) and Iuchi (11) drew an opposite conclusion that patients with EGFR 19del were more likely to develop BMs than patients with L858R mutation. Some studies have failed to show a statistical difference in the association between EGFR mutation subtypes and BM risk (2, 7, 10, 19, 26). The reasons for the significant difference between EGFR 19del and L858R mutation remain uncertain. One possible reason for this finding may be the different clinical characteristics and bio-behaviors of EGFR 19del and L858R mutation. Zhu et al. found that gefitinib inhibited the phosphorylation of EGFR, Akt, and Erk to a greater degree in exon 19 deletion cells than in L858R mutation cells, suggesting that they were two types of NSCLCs (27). This suggests that patients with L858R mutation may more likely to develop BMs than 19del. This controversy may be due to retrospective studies with different baselines and treatments. A further prospective study is needed, and specific mechanisms need to be further discussed.

## Heterogeneous distribution of epidermal growth factor receptor gene mutation

There is discordance in EGFR gene status between paired primary tumors and brain metastatic sites. However, the EGFR gene status of the primary sites can largely represent that of brain metastatic sites. It is sufficient for clinical decision-making, and there is no need to re-biopsy metastatic sites for most NSCLC patients, especially for the metastatic sites that are difficult to access. However, a re-biopsy and a second detection of EGFR mutations are needed if there is a progressive disease and no response to EGFR-TKIs.

Most studies on the gene mutation characteristics of NSCLC are based on primary tumors. There is limited information regarding the spectrum of “druggable” gene abnormalities in CNS metastases of NSCLC. The EGFR mutation status of metastatic tumors does not always coincide with that of the primary sites. Several studies have revealed the heterogeneity of cancer cells and different metastatic sites (28, 29). Gow et al. (30) found that the frequencies of EGFR gene mutations were 27% (18 of 67) in primary lung tumors and 39% (26 of 67) in the corresponding metastases. Nine of 18 (50%) patients with EGFR-mutated primary lung tumors had lost mutations in their metastases. Among the 26 patients who were EGFR mutation-positive for the metastatic tumors, 17 (65%) were negative for the primary tumors. Han et al. (31) explored the agreement of EGFR mutations between the primary and corresponding metastatic tumors (pleural effusion, pleura, brain, lymph node, lung, soft tissue, adrenal gland, etc.). The discordance rate of all patients was 18.9%, and that of patients with mutated EGFR is 35.0%. These data show that primary tumors, the easiest tissues to obtain for patient studies, may not provide a clear representation of the EGFR mutation status of the metastatic tumors and suggest that EGFR mutations may change during metastases.

Given the poor prognosis of brain metastatic lung cancer, there is an urgent need to understand brain metastatic genetic profiles by comparing somatic mutations between primary tumors and corresponding brain metastases, which has important clinical implications. In Caucasians, the average frequency of EGFR mutation in metastatic brain tumors from NSCLC is 6.2% (24/390) (1.6% in 19del and 3.7% in L858R) (**Table 1**) (32–36), which is lower than the EGFR mutation rate reported previously among the non-Asian origin (46, 47). Nicos et al. (36) reported that the EGFR mutation rate of BM sites was 10% for adenocarcinoma, 16.5% for never smokers, and 3% for smokers. Two small-sample studies reported that the concordance rates of EGFR mutation status between paired primary NSCLC and BMs were 100% in all patients (overall concordance) and 100% in mutated patients (mutated concordance) (34, 36). The mean EGFR mutation rate of BMs is 51.5% (**Table 1**) in Asian NSCLC patients, which is similar to that of the primary site (30, 31, 37–45, 48, 49). The average frequency of 19del in BMs is 31.1% (104/334), which is higher than that of L858R (21.0% (70/334)). Wang et al. (43) identified EGFR mutations in 54 paired BMs and primary tumors of NSCLC in synchronous and metachronous BM patients using next-generation sequencing (NGS), the BM/primary concordance rate was 92% (Cohen’s κ coefficient, 0.799), and concordance of patients with mutated EGFR was 85.2% (23/27). In another study, Rau et al. (39) analyzed 49 paired tissues with both primary lung adenocarcinoma and synchronous or metachronous BM lesions. The results revealed a discordance rate of 26.5%. In a study conducted in Korea by Kim et al. (45), the discordance rate between primary NSCLC and metastatic brain lesions was 22.5%. The concordance rates of different studies are summarized in **Table 1**. The combined overall concordance of paired primary and BM sites is 85.6% (154/180) in Asian patients with NSCLC and 100% (38/38) in non-Asian patients (p = 0.01). There is no statistically significant difference in combined mutated concordances between Asian and non-Asian patients with NSCLC (76.0% *vs.* 100%, p = 0.08). A diversity of concordance is also observed in the EGFR subtypes. Wang et al. (43) reported that the concordance in EGFR 19del was 100% (14/14) between BMs and primary lung tumors, whereas a lower concordance rate was also observed in EGFR L858R (71% (5/7)). Interestingly, Han et al. (31) reported a similar result: the discordance in EGFR L858R (50.0%, 5/10) was higher than that in EGFR 19del (22.2%, 2/9). However, the difference between EGFR subtypes was not significant in a study by Gow (30).

**Table 1:** EGFR mutation status in primary tumors and corresponding metastases.

Author	Country	pathology	BM type	Detection methods	EGFR mutation rate of paired tissue			Concordance of paired tissue			EGFR mutation rate of BMs		
					primary	metastasis		all patients	mutated patients		total	19del	L858R
**Europe and America**													
A Kalikaki, 2008 (32)	Greece	ADC, SCC, LCC	NA	direct sequencing	5/25	Metastases: 3/25		19/25 (76.0%)	1/7 (14.3%)		3/25 (12.0%)	1/25 (4.0%)	0/25 (0%)
Delicia Munfus-McCray, 2011 (33)	USA	ADC	NA	Bidirectional sequencing	3/9	Metastases: 2/9		8/9 (88.9%)	2/3 (66.7%)		4/10 (40%)	NA	NA
Claire Villalva, 2013 (34)	France	ADC, SCC, LCC	SC	pyrosequencing	3/8	Brain: 3/8		8/8 (100%)	3/3 (100%)		3/77 (3.9%)	0/77 (0.0%)	3/77 (3.9%)
Wojas-Krawczyk Kamila, 2013 (35)	Poland	ADC, SCC, LCC, others	NA	PNA–LNA PCR clamp assays	NA	NA		NA	NA		9/143 (6.3%)	3/143 (2.1%%)	6/143 (4.2%)
Marcin Nicos, 2018 (36)	Poland	ADC, SCC, LCC, others	SC	direct sequencing	7/30	Brain: 7/30		30/30 (100%)	7/7 (100%)		9/145 (6.2%)	NA	NA
Combined results (results of BMs)								38/38 (100%)	10/10 (100%)		24/390 (6.2%)	4/245 (1.6%)	9/245 (3.7%)
**Asia**													
Shingo Matsumoto, 2006 (37)	Japan	ADC	NA	direct sequencing	6/8	Brain: 6/8		8/8 (100%)	6/6 (100%)		12/19 (63.2%)	10/19 (52.6%)	2/19 (10.5%)
C.-H. Gow, 2009 (30) *	Taiwan	ADC, SCC, LCC, LLC	NA	direct sequencing, SARMS	5/25, 11/25	Brain: 11/25, 11/25		17/25 (68.0%), 22/25 (88.0%)	4/12(33.3%), 10/12 (83.3%)		11/25 (44.0%)	7/25 (28.0%)	4/25 (16.0%)
Hye-Suk Han, 2011 (31)	Korea	ADC	SC	direct sequencing	4/5	Brain: 3/5		4/5 (80%)	3/4 (75%.0)		3/5 (60.0%)	1/5 (20.0%)	2/5 (40.0%)
Dongdong Luo, 2014 (38) **	China	ADC, ASCC, SCC, LCC	NA	SARMS	7/15	Brian: 8/15		14/15 93.3%)	7/8 (87.5%)		72/136 (52.9%)	44/136 (32.4%)	32/136 (23.5%)
Kun-Ming Rau, 2016 (39) **	Taiwan	ADC	SC+MC	SARMS	30/49	Brain: 30/49		36/49 (73.5%)	21/34 (61.8%)		30/49 (61.2%)	17/49 (34.7%)	15/49 (30.6%)
Yun Fan, 2018 (40)	China	ADC	SC+MC	NGS	selected 19del or L858R in primary tumor or CSF.			NA	10/11 (90.9%)		NA	NA	NA
Li Liao, 2018 (41)	China	ADC	SC	NGS	4/6	Brain: 4/6		6/6 (100%)	4/4 (100%)		4/6 (66.7%)	3/6 (50.0%)	1/6 (16.7%)
Yangchun Ma, 2018 (42)	China	ADC	NA	NGS	NA	NA		NA	NA		6/15 (40.0%)	2/15 (13.3%)	4/15 (26.7%)
Hongsheng Wang, 2019 (43)	China	ADC, SCC, others	SC+MC	NGS	24/54	Brain: 25/54		50/54 (92.6%)	23/27 (85.2%)		26/61 (42.6%)	15/61 (24.6%)	7/61 (11.5%)
Ruofan Huang, 2019 (44)	China	ADC	NA	ddPCR	22/34	CSF: 15/34		25/34 (73.5%)	14/23 (60.9%)		NA	NA	NA
Kyung-Min Kim, 2019 (45)	Korea	NA	NA	real-time PCR clamping method	8/18	Brain: 8/18		14/18 (83.8%)	5/9 (66.7%)		8/18 (44.4%)	5/18 (27.8%)	3/18 (16.7%)
Combined results								154/180 (85.6%)	79/104 (76.0%)		172/334 (51.5%)	104/334 (31.1%)	70/334 (21.0%)

*The former data is based on the result only by direct sequencing, and the later by both direct sequnecing and SARMS assay.

** Both 19del and L858R subtype cohorts counts if the patients have double mutation 19del & L858R.

ADC, Adenocarcinoma; SCC, squamous cell carcinoma; LCC, large cell carcinoma; LLC, Lymphoepithelioma-like carcinoma; ASCC, Adenosquamous cell carcinoma; BM, brain metastasis; CSF, cerebrospinal fluid; NA, not available; SC, synchronous, MC, metachronous; PNA–LNA, peptide nucleic acid-locked nucleic acid; SARMS, Scorpion Amplified Refractory Mutation System; NGS, next-generation sequencing; ddPCR, droplet digital polymerase chain reaction (PCR).

“SC+MC” means the BMs developed from diagnosis of NSCLC to the death.

Primary or metastatic CNS malignancies can release trace levels of tumor DNA into the surrounding cerebrospinal fluid (CSF), which could serve as an ideal biomarker for the characterization and monitoring of brain tumors without invasive tissue biopsies, allowing many patients to avoid unnecessary surgery. Huang et al. (44) and Fan et al. (40) detected the EGFR mutation status of the paired primary tumor and corresponding CSF samples from patients with EGFR-mutated lung adenocarcinoma with brain or leptomeningeal metastases. EGFR mutation type(s) in CSF were largely concordant with those in the primary tumor (73.5% concordance for Huang and 90.9% concordance for Fan), proving that EGFR mutation testing in CSF from lung adenocarcinoma patients with CNS metastases is clinically feasible for guiding precision medicine.

When all mutated genes are included in the analysis, this discrepancy is obvious. Wang et al. (43) reported that when considering all driver genes, only 18.0% (11/61) shared the same mutational profiles in primary NSCLC and corresponding brain metastases. Approximately 30% of patients (18 of 61) had brain-unique mutations in addition to those identified in primary lung tumors. Finally, copy number variation (CNV) events of multiple genes, including MET, VEGFA, KEAP1, ROS1, SMAD2, and SMAD4, were found exclusively in BMs. Approximately 13% of the patients (8 of 61) had lung-specific mutations alone. Using whole-exome sequencing, Jiang et al. (50) compared the mutational landscape and evolutionary patterns of lung adenocarcinoma between paired primary lesions (primary lesion of liver metastases (LiM) or BMs) and corresponding metastases. A median shared genetic mutation of 6.8% (range, 0.0%–30.5%) was observed in the BM cohort, which was in sharp contrast to the LiM cohort with a median shared genetic mutation of 66.3% (range, 6.1%–97.1%) (p = 0.005). The data demonstrated that brain metastases presented a far more discrepant mutational landscape than liver metastases when compared with their primary tumors.

No previous studies have reported an association between the EGFR mutation status of metastatic tumors and responsiveness to EGFR-TKIs in NSCLC. Han et al. (31) observed responsiveness to EGFR-TKIs in 18 patients. Two of seven patients with progressive disease who were administered EGFR-TKIs harbored EGFR mutations in the primary tumors but not in metastases, and one of the seven patients who achieved a partial response did not harbor an EGFR mutation in the primary tumor but did have a mutation in the metastasis. This biological phenomenon of discordant EGFR mutations could partially account for the fact that some advanced NSCLC patients with wild-type EGFR respond to EGFR-TKI therapy and why some patients with well-known EGFR-TKI-sensitive mutations fail to respond to EGFR-TKI therapy. Therefore, a re-biopsy of the progression site and a second detection of EGFR mutations are needed.

Generally, cancer cells have difficulty penetrating the blood–brain barrier with the tight layer of endothelial cells (51). Nevertheless, NSCLC cancer cells, especially those with EGFR mutations, target and infiltrate the brain frequently. Considering this metastatic selective advantage, these cancer cells may require highly specialized functions during infiltration into the brain parenchyma. The phenomenon of early and late, multidirectional, monoclonal and polyclonal, and monophyletic and polyphyletic metastatic spread is observed across cancers and within individual cases (52). Macroevolutionary shifts (the onset of chromosomal instability) may lead to more heterogeneous populations of cancer cells and contribute to the evolution of metastatic disease through the genetic draft or clonal selection during tumor progression (52–54). The evolutionary cancer cells acquire metastatic abilities, resulting in different EGFR mutation statuses in primary and metastatic tumors. The clones contain the enhanced metastatic potential of different EGFR mutation statuses from the primary tumors (55). Third, the different microenvironments of primary and metastatic tumors can independently influence the evaluation process of tumor cells, leading to the gain or loss of EGFR mutations (56–59). Finally, the gene mutation detection method and sample quality can give rise to a discrepancy in the EGFR mutation status between the primary site and the metastasis. For example, the sensitivity of direct sequencing is relatively low (30, 60, 61). Due to technical challenges and sample quality, ALK gene detected by fluorescence *in situ* hybridization (FISH) shows more frequent discordances between primary tumor and matched metastases than by immunohistochemistry (60). Cortot et al. (61) used mutant-enriched PCR (ME-PCR) to resolve the discordance in three of six cases of mutant KRAS that were negative by direct sequencing. In a study, Gow et al. (30) applied Scorpion Amplified Refractory Mutation System (SARMS) and direct sequencing to compare EGFR mutations between both sites. The disagreement between paired primary sites and brain metastases decreased from 32% to 12% when a combination of both methods was used as compared to direct DNA sequencing. Patients with mutated EGFR may be misdiagnosed as having no mutations or may be unable to detect major mutations in mixed tumor clones by nucleotide sequencing. Therefore, more sensitive and specific methods or combined methods should be applied to resolve the discrepancies caused by sequencing methods.

## Clinical characteristics of brain metastases

Brain metastases are more common in NSCLC patients with neurological symptoms and advanced-stage patients. Steindl et al. (62) evaluated the incidence, distribution, and impact of neurological symptoms at the time of BM diagnosis. A total of 1,608 patients (529 patients tested for driver gene: 94 (17.8%) with EGFR mutation and 23 (4.3%) with ALK rearrangement) were available for further analyses. Neurological symptoms including focal deficits (985 patients, 61.3%), signs of increased intracranial pressure (483 patients, 30.0%), epileptic seizures (224 patients; 13.9%), and neuropsychological symptoms (233 patients, 14.5%) were documented in 1,186 of 1,608 patients (73.8%). The proportion of symptomatic patients decreased with the advancement of brain metastatic management in recent years (2010–2019) compared to that in the earlier years (1986–1999) (67.8% *vs.* 97.3%, p < 0.001). They also found that the presence of neurological symptoms was irrespective of the number of BMs or the localization of the BMs (p > 0.05, chi-square test), but the size of the BMs at the time of diagnosis was significantly associated with neurological symptoms (p < 0.001). In a study by Hsiao et al. (63), they also reported that larger metastatic brain tumors were associated with CNS symptoms, but in their study, symptomatic patients had a larger number of metastatic brain tumors (4.0 ± 2.1 *vs.* 2.7 ± 1.9, p < 0.001) and multiple BMs (>4 sits) (50% *vs.* 21%, p < 0.001). Dube-Pelletier et al. (64) reported that in stage IV NSCLC with unknown EGFR mutation status, the prevalence of synchronous CNS metastases among patients without CNS symptoms was 32% (47/145). A study by Wasp et al. (65), which included stage I–IV patients with unknown EGFR mutation status, revealed that brain metastases were high in symptomatic patients: 30% (3/10) in stage I, 100% (2/2) in stage II, 85.7% (6/7) in stage III, and 70.8% (17/24) in stage IV. In asymptomatic patients, brain metastases were not found in 17 patients with stage I disease (95% CI: 0–19.5%), nor in 16 patients with stage II disease (95% CI: 0–20.6%), but 4 of 36 patients with stage III disease (11%, 95% CI: 3.1–26.1%) and 9 of 45 patients with stage IV disease (20%, 95% CI: 9.6–34.6%) were affected. Brain metastases are unlikely in patients with early-stage NSCLC (i.e., clinical stages I and II) without central neurologic symptoms. The continued use of neuroimaging in the pretreatment evaluation of clinical stage I patients without central neurological symptoms is not needed.

Among the patients who developed synchronous brain metastases, EGFR mutation-positive cases were less likely to have CNS symptoms than EGFR mutation-negative cases (**Table 2**). However, the possibility of neuro-symptoms is high in patients with metachronous BMs. Ando et al. (67) reported that out of 46 patients with synchronous brain metastasis, 63% (29/46) were asymptomatic and 13% (6/46) patients were clinical stage T1-2aN0. In clinical stage T1-2aN0 cases, only one patient initially presented with neurological symptoms. Furthermore, the symptoms of patients with mutated EGFR were 19.0% (4/21), which was smaller than that of their EGFR-wild counterparts (50.0%, 7/14) (p = 0.049). They also revealed that there were no significant differences in stage T/N and the number of brain metastases between CNS symptom-positive and symptom-negative patients with unknown EGFR mutation status. Sekine et al. (66) reported that the neuro-symptom rates of synchronous BM patients were 25.8% (8/31) in EGFR wild-type patients and 7.7% (2/26) in EGFR-mutated patients. However, a retrospective study demonstrated that there was no significant difference in the incidence of symptomatic BMs at diagnosis (35.4% (34/96) for patients with wild-type EGFR and 31.0% (18/58) for patients with mutated EGFR) (11). As for newly diagnosed BMs during treatment, neuro-symptoms are more common. Steindl et al. (62) explored the incidence of CNS symptoms between initial BMs (confirmed within 30 days of NSCLC diagnosis) and subsequent BMs (>30 days) among patients with unknown EGFR mutation status. Of the 875 patients who presented with initial BMs, 68.2% were neuro-symptomatic, whereas 80.4% of patients (733 cases) with subsequent BMs had CNS symptoms (p < 0.001). Ma et al. (24) reported that among 34 patients (34/134, 25.4%) who developed metachronous BM during EGFR-TKI therapy, the incidence of CNS symptoms was as high as 82.4% (28/34).

**Table 2:** Comparison of CNS symptoms between EGFR mutated-type and EGFR wild-type patients with brain metastases.

Author	Type of BM	EGFR MT	EGFR WT	p-Value
Akimasa Sekine, 2012 (66)	SC	7.7% (2/26)	25.8% (8/31)	0.073
Toshihiko Iuchi, 2015 (11)	SC	31.0% (18/58)	35.4% (34/96)	0.577
Takahiro Ando, 2018 (67)	SC	19.0% (4/21)	50.0% (7/14)	0.049
Xiaoyan Ma, 2016 (24)	MC	82.4% (28/34)	–	–
Min Young Baek, 2018 (9)	SC+MC	51.9% (14/27)	57.5% (23/40)	0.803

“SC+MC” means the BMs developed from diagnosis of NSCLC to death.

SC, synchronous; MC, metachronous; MT, mutated type; WT, wild type; CNS, central nervous system; EGFR, epidermal growth factor receptor; BM, brain metastasis.

## Imaging characteristics of brain metastases

In this part, we discussed heterogeneity of distribution, size, and number of brain metastases. There is no significant difference in the distribution of BMs between EGFR-mutated and EGFR-wild NSCLC. Wang et al. (68) analyzed the distribution of 335 brain metastases from lung cancer. Lung adenocarcinoma exhibited a higher rate of metastasis in the left frontal lobe (53%; 111/208), right frontal lobe (48%; 100/208), and cerebellum (56%; 116/208), with no significant difference in metastasis between these three regions (p > 0.05). In lung squamous cell carcinoma, the cerebellum (70%; 14/20 patients) was the most common site of metastasis (p < 0.05). In patients with EGFR gene mutations, the left frontal lobe (62%; 23/37 patients), right frontal lobe (62%; 23/37 patients), and cerebellum (57%; 21/37 patients) had the highest rate of metastases. However, there was no statistically significant difference in the distribution of patients with wild-type EGFR (26 patients) (p = 0.998). Takano et al. (69) revealed that brain metastases with the EGFR L858R mutation occurred more often in the caudate, cerebellum, and temporal lobe than those with EGFR 19del. Median depths of the lesions from the brain surface were 13.7 mm (range, 8.6–21.9) for EGFR 19del, 11.5 mm (6.6–16.8) for L858R mutation, and 15.0 mm (10.0–20.7) for EGFR-wild type. Lesions with EGFR L858R were located significantly closer to the brain surface than lesions with EGFR 19del or EGFR-wild type (p = 0.0032 and p = 0.0001, respectively). Furthermore, brain metastases of lung adenocarcinoma with a history of chemotherapy but not molecular targeted therapy were located significantly deeper from the brain surface (p = 0.0002).

In most studies, NSCLC BMs with mutated EGFR have smaller sites and showed a trend to have multiple sites when compared to BMs with wild-type EGFR (**Table 3**). In Kim’s research including unselected NSCLC patients with extracranial stage I–IV (21), among 203 patients with synchronous brain metastases, 183 patients (90%) had parenchymal metastases and 42 patients with single parenchymal metastases (23%; mean size, 10.1 ± 9.6 mm), and there were 141 patients with multiple parenchymal metastases (77%; mean size of the largest lesion, 15.8 ± 12.7 mm). Shin et al. (13) reported that among patients with synchronous BM, those with EGFR mutation were more likely to have a larger number of metastatic sites compared to those without EGFR mutations, whereas there was no difference in the size of the largest brain tumors regardless of the EGFR mutation status. Chang et al. (20) reported that the mean BM size of EGFR mutation-positive (N = 280) and mutation-negative (N = 211) was 30.2 and 38.5 mm, respectively (p < 0.001). A higher proportion of patients with mutated EGFR had a tumor size of less than 30 mm (60.4% vs. 43.6%, p < 0.001) and earlier stages (p < 0.001). Iuchi et al. (11), Baek et al. (9), and Han et al. (2) compared the number or size of overall BMs between patients with mutated EGFR and patients with wild-type EGFR: the number of overall BMs showed a trend toward multiple BMs, but only the result of Han et al. had a statistical difference. However, there was an opposite result regarding the size of overall BMs. In Han’s study, patients with mutated EGFR had a larger mean size of BMs compared to patients with wild-type EGFR. However, Iuchi reported that EGFR-positive patients had a smaller median size of BMs. Sekine et al. revealed the influence of the EGFR subtype on the imaging features of BMs from NSCLC (66). Patients with EGFR 19del (18 patients) were more likely to have multiple BMs and smaller brain tumors with smaller peritumoral brain edema than those (31 patients) with wild-type EGFR (p = 0.024, p = 0.0016, and p = 0.0036, respectively). As for patients with EGFR L858R (eight patients), there was no significant difference in any of the values when compared to the patients with wild-type EGFR. In contrast, Yuan et al. (70) compared the patterns of BMs based on early (≤6 months after NSCLC diagnosis) and late (>6 months) presentation. In EGFR wild-type cases (N = 327, 207 early *vs.* 120 late), there was no significant difference in the largest size of BMs, number of metastases, cerebral edema, and leptomeningeal disease. In patients with mutated EGFR with late BMs (103: 65 early *vs.* 38 late), there was a trend toward multiple lesions (46/65 (71%) *vs.* 35/38 (92%), p = 0.012) and leptomeningeal disease (3/207 (1%) *vs.* 10/120 (8%), p = 0.005).

**Table 3:** Number and size of brain metastasis lesions according to EGFR mutation type.

Author	Country	Pathology	Stage	BM type	EGFR status	Total patients	Number	Size (mm)	Others
Akimasa Sekine, 2012 (66)	Japan	ADC, ASCC, and others	IV	SC	EGFR−	31	Median No. 3 (1–38) Multiple (≥2): 23/31 (71.2%)	Median size: 11.0 (2.9–62.0)	PTBE size: 8.8 mm (0.00–76.6 mm)
					19del	18	Median No. 5 (1–100) (p = 0.024 vs. EGFR−) Multiple (≥2): 16/18 (88.9%)	Median size: 7.1 (3.0–20.0) (p = 0.0016 vs. EGFR−)	PTBE size: 0.3 mm (0.00–36.6 mm) (p = 0.0036 vs. EGFR−)
					L858R	8	Median No. 4 (1–20) Multiple (≥2): 6/8 (75%)	Median size: 10.0 (3.2–41.0)	PTBE size: 6.1 mm (0.00–57.0 mm)
Dong-Yeop Shin, 2014 (13)	Korea	ADC	NA	SC	EGFR+	33	Median No. 5 (range 1–57) Multiple (≥2): 78.6%	Size of the largest brain tumors: 1.2 (0.3–4.3)	–
					EGFR−	18	Median No. 2 (range 1–21) (p = 0.029) Multiple (≥2): 47.8% (p = 0.022)	Size of the largest brain tumors: 0.9 (0.4–5.0) p = 0.44	–
Toshihiko Iuchi, 2015 (11)	Japan	ADC, SCC, and others	I–IV	SC+MC	EGFR+	93	Single (1): 33/93 (35.5%) Oligo (2–4): 27/93 (29.0%) Multiple (≥5): 33/93 (35.5%)	Median size: 9.1 (0.1–47.2)	–
					EGFR−	152	Single (1): 67/152 (43.5%) Oligo (2–4): 45/152 (29.3%) Multiple (≥5): 40/152 (27.3%) p = 0.262	Median size: 11.7 (2–139.5) p = 0.031	–
Ren Yuan, 2016 (70) *	Canada	Non-SCC NSCLC	IV	Early (≤6 months) vs. late (>6 months)	EGFR+	103 (65 early vs. 38 late)	Multiple (≥2): 46/65 (71%) vs. 35/38 (92%), p = 0.012	Median size of largest: 18.5 (4–59) vs. 16.5 (5–51), p = 0.7	Edema: 186/207 (90%) vs. 108/120 (90%), p = 0.9 Leptomeningeal disease: 3/207 (1%) vs. 10/120 (8%), p = 0.005
					EGFR−	327 (207 early vs. 120 late)	Multiple (≥2): 128/207 (61%) vs. 75/120 (62%), p = 1	Median size of largest BM: 21 (4–90) vs. 20 (5–60), p = 0.5	Edema: 52/65 (80%) vs. 27/38 (71%), p = 0.4 Leptomeningeal disease: 7/65 (11%) vs. 4/38 (11%), p = 1
Guang Han, 2016 (2)	China	ADC	I–IV	SC+MC	EGFR+	48	No. 6.85 ± 1.235	Size of largest brain tumors: 19.7 ± 1.39	–
					EGFR−	28	No. 3.43 ± 0.594, p = 0.045	Size of largest brain tumors: 12.1 ± 1.75, p = 0.001	–
Min Young Baek, 2018 (66)	Korea	ADC, SCC, LCC, and others	NA	SC+MC	EGFR+	27	Single (1): 6/27 (22.2%) Oligo (2–4): 5/27 (18.5%) Multiple (≥5): 16/27 (59.3%)	NA	–
					EGFR−	40	Single (1): 13/40 (32.5%) Oligo (2–4): 6/40 (15.0%) Multiple (≥5): 21/40 (52.5%) p = 0.741	NA	–
Wei-Yuan Chang, 2018 (20)	Taiwan	ADC, SCC, and others	I–III	MC	EGFR+	280	NA	Mean size: 30.2 ≤30: 169/280 (60.4%); >30: 109/280 (38.9%)	–
					EGFR−	211	NA	Mean size: 38.5 ≤30: 92/211 (43.6%); >30: 115/211 (54.5%) (p < 0.001 *vs.* EGFR+)	–
Takahiro Ando, 2018 (67)	Japan	ADC	IV	NA	EGFR+	21	Multiple (≥2): 16/21 (76.2%)	NA	–
			IV		EGFR−	14	Multiple (≥2): 10/14 (71.4%), p = 0.71	NA	–
Minjae Kim, 2020 (21)	Korea	ADC, SCC, and others	I–IV	SC	NA	203	Single (1): 42/183 (23%) Multiple (≥2): 141/183 (77%)	Mean size: 10.1 ± 9.6 Mean size of largest: 15.8 ± 12.7	183 patients (90%) had parenchymal metastases

PTBE, peritumoral brain edema; ADC, adenocarcinoma; SCC, squamous cell carcinoma; ASCC, adenosquamous cell carcinoma; LCC, large cell carcinoma; EGFR, epidermal growth factor receptor; EGFR+, mutated EGFR; EGFR−, wild-type EGFR; SC, synchronous; MC, metachronous; BM, brain metastasis; NA, not available.

*“Early” means the BMs developed less than or equal to 6 months after NSCLC diagnosis, and “late” means the BMs developed over 6 months.

For very early-stage NSCLC patients, especially those without neuro-symptoms, regular follow-up (RFU) of neuroimaging is not recommended. Regarding the timing of neuroimaging in advanced NSCLC patients with EGFR mutation, liberal follow-up (LFU) of brain imaging at first several years showed more advantages in terms of cost. The rationale behind routine neuroimaging in patients with advanced disease is that the early detection of brain metastases can lead to early treatment before the development of neurologic deficits or seizures. Considering the improved OS among BM patients with easy access to EGFR-TKIs and the burden of the healthcare system, brain surveillance strategies have become very important (71–76). In the study by Wasp et al. (65), which included stage I–IV patients with unknown EGFR mutation status, initial neuroimaging was performed in 72.7% (157/216) of the patients. No brain metastases were found in stage I–II patients without CNS symptoms. However, brain metastases were much higher in symptomatic patients. Kim et al. (21) suggested that a particularly low BM incidence in cIA disease (0.3%, 2/615) provides evidence that there is no need for staging brain MRI in patients with cIA NSCLC, whereas brain MRI should be considered in stage over cIA disease or EGFR-mutated adenocarcinoma. A retrospective study showed that the frequency of asymptomatic brain metastasis in patients with stage I–III NSCLC was 5.7% (77). Enlargement of lymph nodes was the only predictor of asymptomatic brain metastases, indicating over-utilization of MRI in early-stage disease, especially in NSCLC patients without lymph node metastases. In a unicentric retrospective study, CNS imaging was performed at diagnosis in 56% (145/257) of patients with stage IV NSCLC, and there was no benefit with routine neuroimaging in terms of median OS (with initial neuro-imaging *vs.* without initial neuro-imaging: 5.9 months (95% CI: 4.0–7.8) *vs.* 5.8 months (95% CI: 4.1–7.1) in patients without neurologic symptoms (64). Shen et al. (78) evaluated the effects of two different brain surveillance strategies: RFU and LFU. A total of 310 stage IV patients with mutated EGFR were included, 43.5% of whom initially had brain metastases. The overall survival and median time-to-treatment failure (RFU = 12.6 months, LFU = 12.4 months; p = 0.924) of patients with initial EGFR-TKI treatment showed no statistical difference between the two groups. However, the cost of brain imaging per patient per year before intracranial progression was higher in the RFU group (approximately 1575 USD) than in the LFU group (approximately 676 USD) (p < 0.001). For patients with EGFR-mutated lung adenocarcinoma who used EGFR-TKIs as frontline therapy, liberal brain MRI follow-up showed more advantages in terms of cost. Mitra et al. (6) performed a competing risk analysis of brain metastasis in locally advanced NSCLC with EGFR mutations. The study indicated that the risk of BMs increases most rapidly from 1 year after initiating definitive therapy for 5 years. As a result, they suggested surveillance of brain MRI every 6 months, beginning a year after treatment initiation and ending 5 years later. Another retrospective multivariable logistic regression analysis showed that the rise in the cumulative incidence curve of metachronous BMs of EGFR-mutated cases reached a plateau 3 years after the diagnosis of lung cancer (10). Surveillance brain MRI is recommended for EGFR-mutated cohorts, especially during the first 3-year follow-up.

## Influence of treatments on brain metastases

During the past decade, the advancement of EGFR-TKIs revolutionarily transformed the treatment landscape of NSCLC with EGFR mutation. Both second-generation EGFR-TKI (afatinib) and third-generation EGFR-TKI (osimertinib) delay the development of brain metastases but may be unable to prevent them. Nevertheless, first-generation EGFR-TKI does not show a certain preventive efficiency. Whether there is an association between the timing of EGFR-TKI treatment (first-line, second-line, and third-line or multi-line treatments) and BM development, only one study suggested that third- or multi-line treatment may result in more brain metastases (24). Prophylactic cranial irradiation (PCI) may result in over-treatment as it cannot bring OS benefit.

**Figure 1E** shows the TTBM between patients with mutated EGFR and patients with wild-type EGFR. The results of Heon et al. (79) and Stanic et al. (12) revealed that patients with mutated EGFR had a longer median TTBM than patients with wild-type EGFR, which demonstrated that EGFR-TKIs had a preventive effect against BM. Beak et al. (9) and Yuan et al. (70) reported no significant differences in median TTBM between the two cohorts. However, there was a trend for patients with mutated EGFR to have a longer median TTBM. However, Mitra et al. (6) and Hsiao et al. (10) drew the opposite conclusions.

Studies on the influence of EGFR-TKIs on BM development have been conflicting. Xu et al. (80) estimated the preventive efficacy of EGFR-TKI (gefitinib) and chemotherapy (vinorelbine plus cisplatin) after surgery among stage II–IIIA patients with mutated EGFR. There was no significant difference in the cumulative rates of metachronous BMs (epidermal growth factor receptor EGFR-TKI *vs.* chemotherapy, 29/106 (27.4%) *vs.* 21/87 (24.1%), p = 0.611), although EGFR-TKI treatment significantly improved disease-free survival (EGFR-TKI *vs.* chemotherapy, 28.7 months (95% CI: 24.94–32.46) *vs.* 18 months (95% CI: 13.59–22.34), p = 0.0054; 3-year DFS, 34% *vs.* 27%). Ma et al. (7) investigated the incidence of BMs in patients with EGFR-mutated NSCLC who received chemotherapy and EGFR-TKIs as the first-line treatment. In univariate analyses, patients with mutated EGFR who received first-generation EGFR-TKIs had a low risk of BM development compared to patients who received chemotherapy (HR = 2.296, 95% CI: 1.050-50.18), p = 0.037), whereas no statistical difference was observed in multivariate analyses (HR = 0.504, 95% CI: 0.153-1.660, p = 0.260). The results of Heon suggested that gefitinib and erlotinib may play a role in the prevention of CNS metastases from NSCLC (79). The 6-, 12-, and 24-month cumulative rates of CNS progression were 1%, 6%, and 21%, respectively, in the EGFR-TKI group compared with corresponding rates of 7%, 19%, and 32%, respectively, in the chemotherapy group (HR = 0.56, 95% CI: 0.34–0.94, p = 0.026). The median time to CNS progression was 56.0 months in the EGFR-TKI group and 31.6 months in the chemotherapy group (p = 0.01). Hsiao et al. (10) drew the opposite conclusion. Of the stage IIIB-IV NSCLC patients without BM at the time of diagnosis, 105 patients had EGFR mutations, 33 were treated with EGFR-TKIs (TKI group), and 72 were treated with a non-TKI regimen (non-TKI group) as first-line treatment. More metachronous BM was observed in the TKI group (54.5%, 18/33) than in the non-TKI group (29.2%, 21/72) (HR = 2.10, 95% CI: 1.15-3.82, p = 0.015). Possible reasons for the differences were selection bias, different research methodology, imbalance in EGFR-TKI use, and follow-up time (38 vs. 11.8 months). Another reason may be that first-generation EGFR-TKIs were used in most studies, and the treatment effect on BMs is limited.

Although first-generation EGFR-TKIs have been reported to pass through the blood–brain barrier (BBB) and are effective for EGFR-mutated NSCLC patients with BMs, CNS metastasis represents one of the most common types of EGFR-TKI treatment failure (81–85). Lee et al. (86) found that 26% of patients developed CNS failure and 13% experienced isolated CNS failure among 166 patients with a clinical benefit to first-generation EGFR-TKI (gefitinib or erlotinib) treatment. First-generation EGFR-TKI therapy showed good efficacy in non-BM lesions but had a limited impact on BMs. The serum concentration of TKIs is important to overcome incomplete BBB penetration and effectively control cancer cells with EGFR mutations in the brain (87). The lower CNS concentration of gefitinib than erlotinib may explain the advantage of erlotinib over gefitinib (88, 89). Ouyang et al. (7) reported that different first-generation EGFR-TKIs had no significant differences in their impact on BM development during treatment (gefitinib vs. erlotinib *vs.* icotinib, 19/99 (19.2%) vs. 7/38 (18.4%) vs. 4/20 (20%)). Li et al. obtained similar results (90). Among the patients without initial BM, the median TTBM of erlotinib (n = 84) and gefitinib (n = 149) were similar (18 *vs.* 16 months, p = 0.392). However, among the patients with initial BM, the median time to CNS progression was 30 months for erlotinib (n = 24) and 15.8 months for gefitinib (n = 22) (p = 0.024).

In a combined analysis of LUX-Lung 3 and 6 trials, first-line afatinib (a second-generation EGFR-TKI) significantly improved progression-free survival (PFS) (8.2 *vs.* 5.4 months; HR = 0.50, p = 0.03) and objective response rate versus chemotherapy in NSCLC patients with brain metastases and common EGFR mutations (91). Yang et al. (92) reported that the 6- and 12-month cumulative rates of metachronous BMs in patients receiving afatinib were 1.3% and 2.6%, respectively. Su et al. (4) evaluated the efficacy of three first-line EGFR-TKIs in preventing and treating BMs and proved that afatinib tended to provide better BM prevention than gefitinib (HR 0.49; 95% CI: 0.34–0.71, p < 0.001) after using Cox proportional hazards regression to adjust for possible confounders. No significant difference was observed between gefitinib and erlotinib treatments. However, the three curves of cumulative rates of metachronous BM separated well from each other until approximately 24 months (CRs of metachronous BM at 6, 12, 24, and 36 months: gefitinib group, 3.8%, 13.9%, 34.6%, and 53.6%; erlotinib group, 5.6%, 9.3%, 9.3%, and 60.3%; afatinib group, 0%, 2.8%, 28.3%, and 41.5%, respectively).

A large-scale real-world study compared the efficacy of first- and third-generation EGFR-TKIs in preventing or delaying symptomatic and metachronous CNS metastasis among patients with mutated EGFR with advanced NSCLC and those without baseline CNS lesions (25). In this study, a lower cumulative incidence of CNS metastasis was observed among patients receiving osimertinib than among those receiving first-generation EGFR-TKIs (p = 0.053). However, the curves of symptomatic CNS metastasis between the first-generation EGFR-TKI group and the osimertinib group tended to reach a plateau after approximately 3 years, and the cumulative incidence of CNS metastasis beyond this period was similar. The 3-year cumulative rates of symptomatic CNS metastasis were 15.6% for first-generation EGFR-TKIs (gefitinib plus erlotinib) and 11.8% for osimertinib. Moreover, the advantage of osimertinib in delaying symptomatic CNS metastasis appeared to be more profound in patients with the L858R mutation (p = 0.053) than in those patients with the 19del mutation (p = 0.744). Osimertinib demonstrated a higher penetration of the BBB than gefitinib and afatinib, which can explain its preventive effect on BMs (93, 94). These data indicate that osimertinib delays the development of symptomatic CNS metastasis but may be unable to prevent it.

There are insufficient studies available regarding the treatment timing of EGFR-TKIs, especially in advanced NSCLC with BM. Koo et al. (95) reported that EGFR-TKIs showed similar efficacy in EGFR-mutated lung adenocarcinoma in terms of response rate, PFS, and OS regardless of the treatment timing. Among the patients with stage IIIB–IV disease who received EGFR-TKI therapy in the study by Ma et al. (24), 20.2%, 53.7%, and 26.1% of patients received EGFR-TKIs as first-line, second-line, and third-line or multi-line treatments, respectively. Univariate analysis indicated that patients with third- or multi-line treatment were more likely to develop metachronous BMs (p = 0.025), whereas multivariate analysis failed to show a statistical difference in the association between the timing of EGFR-TKI treatment and BM development (p = 0.401). Whether there were some differences among first-line, second-line, and third-line or multi-line treatments in terms of BM development needs further exploration.

Previous phase III studies have looked at the role of PCI versus observation in radically treated unselected stage III NSCLC patients without initial CNS involvement (96–99). Although PCI decreased the risk of BM development, no improvement in the OS was observed. Furthermore, it is well documented that PCI is accompanied by the potential of significant neurocognitive toxicity (100, 101). Given the lack of OS benefit and unknown efficacy in patients with mutated EGFR receiving EGFR-TKI treatment, PCI would still likely result in over-treatment. This is particularly true in the era in which third-generation EGFR-TKIs are easily accessible, and stereotactic radiation therapy can often be effective while sparing neurocognitive function.

## Prognosis of patients with brain metastases

With the advancement in EGFR-TKIs, the prognosis of EGFR-mutated NSCLC has improved significantly. Compared to EGFR wild-type patients, EGFR-mutated patients with synchronous or early BMs had a longer BMOS. Among NSCLC patients with mutated EGFR, patients who did not develop BMs had a longer OS than those who did. Combined treatments of radiotherapy and EGFR-TKI may further improve OS. The WJTOG3405 trial reported that the median OS of advanced NSCLC patients with mutated EGFR treated with first-generation EGFR-TKIs was up to 30.2 months, which is much longer than that of patients treated with chemotherapy (102). Among synchronous or overall BM patients, longer BMOS was observed in patients with mutated EGFR than in patients with wild-type EGFR (**Figure 1F**) (2, 3, 6, 9, 12, 20). Although a trend of longer BMOS was observed among metachronous patients (6, 9, 20), just the result of Mitra et al. was statistically significant (6). It is possible that the small sample size in Baek’s and Chang’s study contributed to the failure to obtain a statistically significant result (9, 20). Furthermore, Li et al. (90) revealed that patients treated with erlotinib had a trend of longer BMOS than those treated with gefitinib, but the difference was not statistically significant (16 *vs.* 12.6 months, p = 0.793). Chang et al. (20) reported that patients with EGFR 19del tended to have longer survival after BM than those with non-EGFR 19del (BMOS, 29.4 *vs.* 14.3 months, HR 0.58, 95% CI: 0.32–1.09, p = 0.089). The deterrence of BM has become an increasingly relevant therapeutic dilemma leading to a poor prognosis. Among advanced NSCLC patients with mutated EGFR and without synchronous BMs, Ouyang et al. reported that patients who did not develop metachronous BMs had a much longer median overall survival than those who did (44.8 vs. 22.1 months, p = 0.027). In another retrospective study by Su et al. (4), they demonstrated that the stage IIIB–IV patients with mutated EGFR without synchronous BMs had a better PFS (12.2 *vs.* 8.9 months, p < 0.001) and OS (35.5 *vs.* 22.1 months, p = 0.015) than those with synchronous BM. Yuan et al. evaluated the differences between early (≤6 months after diagnosis) and late (>6 months) BMs. In early BMs, patients with mutated EGFR had a survival comparable to late BMs (19.9 *vs.* 25.6 months). In contrast, EGFR wild-type patients with early BMs have a significantly reduced OS than those with late BMs (7.1 *vs.* 24.9 months). Further studies demonstrated that concurrent chemoradiotherapy followed by EGFR-TKI led to a better median OS, ranging from 46.9 to 57.9 months (16–18). Recently, Akamatsu et al. (103) combined radiotherapy with gefitinib concurrently to treat similar patients, and the median OS was prolonged to 61.1 months (95% CI: 38.1 months—not reached).

## Conclusion

The characteristics of NSCLC BMs in patients with mutated EGFR and patients with wild-type EGFR differ significantly. This review discussed the incidence of BMs, clinical and imaging characteristics of brain metastases, brain surveillance strategies, influence of treatments on BMs, prognosis after BMs, and difference in EGFR mutations between paired primary tumors and BMs in EGFR-mutated NSCLC. We expect that this will help in the management of brain metastases.

## Author contributions

WZa and WZo were the main writers of the review and completed the collection and analysis of the relevant literature and the writing of the first draft of the paper. LR, MS, XL, LW and SW participated in the analysis and collection of the data and revised the draft. YW and ZH were the designers and in charge of the project and directed the article writing. All authors contributed to the article and approved the submitted version.

## Funding

This work is supported by Integrated innovation and application of key technologies for precision prevention and treatment of primary lung cancer (2019ZX002).

## Conflict of interest

The authors declare that the research was conducted in the absence of any commercial or financial relationships that could be construed as a potential conflict of interest.

## Publisher’s note

All claims expressed in this article are solely those of the authors and do not necessarily represent those of their affiliated organizations, or those of the publisher, the editors and the reviewers. Any product that may be evaluated in this article, or claim that may be made by its manufacturer, is not guaranteed or endorsed by the publisher.
